# Preparing learners with partly incorrect intuitive prior knowledge for learning

**DOI:** 10.3389/fpsyg.2014.00664

**Published:** 2014-07-03

**Authors:** Andrea Ohst, Béatrice M. E. Fondu, Inga Glogger, Matthias Nückles, Alexander Renkl

**Affiliations:** ^1^Department of Educational and Developmental Psychology, University of FreiburgFreiburg, Germany; ^2^Department of Educational Sciences, University of FreiburgFreiburg, Germany

**Keywords:** knowledge in pieces, generalized schemata, teacher education, learning strategies, cognitive load theory

## Abstract

Learners sometimes have incoherent and fragmented intuitive prior knowledge that is (partly) “incompatible” with the to-be-learned contents. Such knowledge in pieces can cause conceptual disorientation and cognitive overload while learning. We hypothesized that a pre-training intervention providing a generalized schema as a structuring framework for such knowledge in pieces would support (re)organizing-processes of prior knowledge and thus reduce unnecessary cognitive load during subsequent learning. Fifty-six student teachers participated in the experiment. A framework group underwent a pre-training intervention providing a generalized, categorical schema for categorizing primary learning strategies and related but different strategies as a cognitive framework for (re-)organizing their prior knowledge. Our control group received comparable factual information but no framework. Afterwards, all participants learned about primary learning strategies. The framework group claimed to possess higher levels of interest and self-efficacy, achieved higher learning outcomes, and learned more efficiently. Hence, providing a categorical framework can help overcome the barrier of incorrect prior knowledge in pieces.

## Introduction

Prior knowledge is usually very helpful in acquiring further knowledge. However, when learners have prior knowledge that is incompatible with the scientific knowledge to be learned, prior knowledge hinders learning. We found that student teachers often have such partially incorrect and fragmented knowledge about learning strategies (in the sense of Weinstein and Mayer, 1986). When we asked them what they think that learning strategies are, one of their answers was “group work.” Group work, however, occurs at the teacher's initiative, and is therefore not a learning but a teaching strategy. Learning strategies refer to mental processes that are learner-initiated and help construct knowledge (Weinstein and Mayer, 1986; Weinstein et al., 2000).

Learning strategies such as organizing and elaborating play a central role in sustainable learning (e.g., Weinstein and Mayer, 1986; Leutner et al., 2007; Glogger et al., 2012). Therefore, teachers should be able to assess and foster students' learning strategies. Assessing learning strategies requires profound professional knowledge about them (Robertson, 1990; Jonassen, 2000; Glogger et al., 2012). Teachers' lack of knowledge about self-regulated learning and, more specifically, about learning strategies is assumed to be responsible (Dignath et al., 2008) for the observation that teachers neglect learning strategies in their classrooms (McCormick et al., 1989; Clift et al., 1990; Moely et al., 1992, 1995; Rosenshine, 1997; Hamman, 1998; Hamman et al., 2000; Annevirta and Vauras, 2001; Perry et al., 2004).

This is the context in which we instructed student teachers in learning strategies using a computer-based learning environment (Glogger et al., 2013). However, even after instruction, some student teachers still stuck to their prior assumptions and considered that “group work,” for example, was a learning strategy (Glogger et al., 2009). We assume that their learning processes were hampered by unstructured, partly incorrect prior knowledge. Hence, the instruction by Glogger et al. (2009); Glogger et al. (2013) may have been insufficient to alter the incorrect intuitive knowledge. Such incorrect knowledge has recently been increasingly conceptualized within a *knowledge-in-pieces* framework (Özdemir and Clark, 2007; Ozdemir, 2013).

Knowledge in pieces is defined as incoherent, unsystematic, intuitive knowledge which (partly) differs from normative knowledge (diSessa, 1993). These pieces of knowledge can be somewhat compared to puzzle pieces. Imagine you have a pile of pieces from different puzzles in front of you. The individual pieces are unassembled, not sorted according to the puzzle they belong to, and you have no idea how each puzzle should look. Once you obtain a new piece of a particular puzzle you might struggle to put it in the right puzzle and in the right place. A learner with prior knowledge in pieces might likewise struggle to integrate new information about a certain concept. We will refer to this struggle as *conceptual cognitive disorientation*. The processes involved in resolving the integration require *cognitive resources*, which may result in *cognitive overload* and subsequently the failure to learn. Knowledge in pieces can therefore hamper the effectiveness of later instructional lessons.

A pre-training intervention providing a generalized schema as a structuring framework for the given knowledge in pieces could help learners to (re)organize their prior knowledge and thus reduce *intrinsic cognitive load* during subsequent learning (see also Kalyuga et al., 2010). Returning to our analogy, this would mean aligning the pieces of the different puzzles to their respective puzzle (concept) so that new puzzle pieces can be more easily integrated. In the present study we developed just such a pre-training intervention and tested its effects on learning outcome measures. In an initial study, we found preliminary evidence that a cognitive framework provided during pre-training (a) supports more efficient learning (roughly equal learning outcomes in less learning time) and (b) strengthens learners' interest in the topic (Ohst et al., submitted manuscript).

### Strategies to lower intrinsic cognitive load

Load due to the complexity of learning contents in relation to the learners' prior knowledge is referred to as intrinsic cognitive load. *Intrinsic cognitive load* is caused by the learning material itself (Sweller and Chandler, 1994; Sweller, 2010). Very complex material has *high element interactivity*; this means that a learner must consider many elements at the same time, requiring considerable working memory. If a learner has not developed appropriate schemas to process these elements efficiently (*chunking*), high element interactivity can exceed working memory capacity. There are several effective strategies to lower high intrinsic cognitive load (see Ayres, 2013). One is to not present a complex task at the outset but to first reorganize the material and present it as *isolated elements* so that learners can process it sequentially in working memory [e.g., when learning about an electrical safety test, focusing first on explaining only basic procedural steps versus providing all the information at once (Pollock et al., 2002)]. Another strategy is to increase the learner's prior knowledge, for example, in a *pre-training intervention*. Learners can use high prior knowledge to chunk information into larger units, thus reducing intrinsic load [e.g., learners who received a pre-training providing names and behaviors of the component parts could afterwards pay more attention to causal effects and therefore learned more than when learning the component and causal model simultaneously (Mayer et al., 2002)]. However, approaches inspired by cognitive-load research usually do not consider that learners might have prior knowledge that is (partly) “incorrect.” With our student teachers we found that it did not suffice to merely increase their knowledge via a training intervention in learning strategies just to fill their knowledge gap. We assume that their prior knowledge's structure prevented “smooth” knowledge acquisition. In this case, it is not enough to merely reorganize the material by isolating elements or increasing prior knowledge; instead, prior knowledge must be changed. However, intuitive concepts can be resistant to change (see research on *conceptual change*). Hence, learners with incorrect intuitive knowledge should undergo a pre-training intervention that supports knowledge restructuring and prepares them for learning. In order to develop such a pre-training intervention we firstly had to analyze how intuitive knowledge is structured and why it was an obstacle for knowledge acquisition.

### Student teachers' knowledge conceptualized as knowledge in pieces

Intuitive knowledge can be understood as rather unstructured, fragmented, knowledge in pieces(e.g., diSessa, 1993; Wagner, 2006). People sometimes generate such pieces of knowledge as brief heuristics (e.g., for explanation) through daily experiences when they observe different phenomena. Knowledge in pieces is activated *context-sensitively* (Ashe and Bibi, 2011). Pieces of knowledge have a specific *cuing priority* in specific contexts which determines their activation. Single pieces of knowledge are not interconnected, embedded in a framework or form an overarching theory; they remain *isolated* (diSessa, 2002). Due to this fact, contradictory pieces of knowledge can *coexist* without learners' being aware of the contradictions. Thus, in different contexts, different sets of knowledge in pieces become activated and *incoherence* across different contexts characterizes the knowledge in pieces.

In recent years, teachers' “incorrect” intuitive knowledge has been increasingly conceptualized as *pedagogical knowledge in pieces* (Ashe and Bibi, 2011; Kali et al., 2011; Hopper et al., 2012; Orrill and Eriksen Brown, 2012; Harlow et al., 2013). Various studies identified several characteristics of knowledge in pieces in teachers' pedagogical knowledge: (a) Context sensitivity: teachers were sometimes unable to rely on knowledge available in other situations (e.g., Carraher et al., 1985; Kali et al., 2011; Orrill and Eriksen Brown, 2012). (b) Incoherence: teachers' thoughts about one specific teaching situation contradicted those about another specific situation (Kane et al., 2002; Eley, 2006; Postareff et al., 2008). In addition, teachers' beliefs about teaching (e.g., constructivist view of learning) were not in line with their practice (e.g., expecting that every student possesses the same knowledge) (Lee et al., 2013). (c) Prioritization: Clift et al. (1990) asked teachers to select suitable learning strategies from a list for different tasks. The teachers tended to suggest strategies based on superficial information processing rather than on deep processing (such as elaboration). One can interpret this finding as mal-prioritization of their knowledge.

We analyzed teachers' knowledge about learning strategies and can describe several characteristics knowledge in pieces (Glogger et al., 2011, 2012, 2014). As an example of scientific knowledge we refer to frequently cited taxonomies such as that by Weinstein and Mayer (1986) and related approaches. They maintain that a scientifically accepted, correct concept of primary learning strategies should include the following characteristics: learning strategies refer to mental processes that are learner-initiated and help construct knowledge. The term “learning strategies” is sometimes also used to refer to strategies that support learning (e.g., creating a timetable). In order to distinguish learning strategies that are primarily related to the learning processes from those strategies, we will henceforth apply the term “primary learning strategies” to describe those that are learner-initiated and “secondary learning strategies” for those that support learning. Glogger et al. found that student teachers hold pieces of knowledge that are compatible with scientific knowledge (e.g., “elaboration is a primary learning strategy”) as well as pieces of knowledge incompatible with scientific knowledge (e.g., “group work is a primary learning strategy”). They seemed to lack a well-defined concept of primary learning strategies, but rather constructed it on-the-fly. Their concept had fuzzy boundaries and interfered with similar but distinct concepts.

### Addressing teachers' pedagogical knowledge in pieces

In previous studies (Glogger et al., 2009, 2013), it did not suffice to simply provide student teachers with more information about primary learning strategies. Hence we developed a pre-training intervention to support their learning processes. When learners with knowledge in pieces obtain new information (e.g., in a learning environment about primary learning strategies) they may not integrate this new information due to cognitive disorientation and high cognitive load or even overload, as described above. An intervention that both lowers high intrinsic cognitive load and reorganizes such learners' knowledge would make sense. According to the knowledge-in-pieces framework, loosely-connected prior knowledge in pieces should become a better organized, stronger and complex knowledge system (diSessa, 2002; Ozdemir, 2013).

The learners should *reprioritize* and refine their prior knowledge and *displace* inappropriate pieces of knowledge into better fitting categories (diSessa and Wagner, 2005; Ozdemir, 2013). The student teachers seemed to have a mis-assignment problem. In order to develop an appropriate category of primary learning strategies, the student teachers therefore may need to learn what primary learning strategies specifically are (internal boundary) and how to discriminate primary learning strategies (external boundary). Our idea was to provide learners with an abstract categorical scheme in a pre-training intervention (Kalyuga et al., 2010; Kalyuga and Hanham, 2011; Kalyuga, 2013). This pre-training intervention gave them an idea of how to distinguish primary learning strategies from the four similar but distinct categories (secondary learning strategies, problem-solving strategies, using tools, and teaching strategies). The student teachers could use this framework to reorganize their knowledge in pieces and to make the limits of primary learning strategies more salient. Thanks to having access to information about the similar categories, they could incorporate inappropriate pieces of knowledge into better fitting categories (displacement) and thus imagine how to distinguish primary learning strategies from the four similar categories. This schema could be used as a cognitive framework for reorganizing prior knowledge so that new information can be more easily integrated.

In an initial study, we had already tested the effectiveness of such an abstract categorical scheme as a cognitive framework (Ohst et al., submitted manuscript). In that study, the framework group that received an abstract, categorical scheme as a cognitive framework saved a significant amount of learning time compared to the control group and thus learned more efficiently (strong effect). Furthermore, the framework group claimed to possess a significantly higher level of interest in primary learning strategies (medium effect). However, we did detect no significant effect on learning outcomes. One of the study's limitations was that the student teachers devoted very little time to the pre-training intervention (12 slides). Instead of the estimated 15 min (as we had observed in pilot tests), the student teachers inspected the slides for an average of only three-and-half minutes. We assume that the student teachers processed the pre-training contents superficially, meaning that this intervention had little effect (i.e., that is, no effect on learning outcomes). We assumed that an optimized training intervention would trigger effects on learning outcomes. To counteract superficial processing of the complex pre-training intervention in the present (subsequent) study, we interspersed questions in the pre-training intervention that prompted deeper processing (e.g., Schworm and Renkl, 2006).

### Motivational aspects

When teachers are expected to apply knowledge, the feeling of having learned something valuable is important: high levels of self-efficacy create a positive condition for transfer in practice (Gegenfurtner, 2011). Self-efficacy is conceptualized as one's beliefs about one's own capability to perform a particular task (Bandura, 1986). In addition, interest in the learned contents also fosters transfer (Pugh and Bergin, 2006). Both interest and self-efficacy can be fostered by helping learners gain more and more coherent knowledge. Learners' topic-specific interest increased when their initially fragmented bits of knowledge became broader, deeper, and more coherent (Alexander et al., 1994) so that that knowledge became a valuable mental resource (Hidi, 1990). Furthermore, according to interest theories, knowledge sufficient to organize new information is a pre-condition for interest (e.g., Renninger, 2000).

Increased knowledge also enhances self-efficacy. Beliefs of self-efficacy originate through enactive mastery experiences, namely experiences of success or failure in a specific domain (Bandura, 1997; Williams and Williams, 2010; Sitzmann and Yeo, 2013). Enhanced prior knowledge can lead to such a mastery experience and is positively related to self-efficacy (Moos and Azevedo, 2009; Ineson et al., 2013). Furthermore, studies have found a link between expanding knowledge and the enhancement of self-efficacy (e.g., Pajares, 1996; Schunk, 1996; Bandura, 1997; Schunk and Pajares, 2002; Ineson et al., 2013; Wäschle et al., 2014).

### Research questions—hypotheses

We assumed that providing a generalized categorical scheme helped student teachers to reorganize their knowledge and integrate new information. We thus derived the following eight hypotheses. We assumed that the pre-training intervention increased the student teachers' knowledge about learning strategies and enabled them to better understand what primary learning strategies are. Therefore, we expected that the framework group provided a more correct definition for primary learning strategies after working with the learning environment (*Definition Hypothesis*). We also expected that the framework group revealed better learning outcomes on learning strategies. More specifically, we anticipated that the framework group was able to identify more primary learning strategies correctly (*Identification Hypothesis*). Furthermore, we assumed that the pre-training intervention leaded to deeper understanding and consequently to more sophisticated explanations of learning strategies (*Explanation Hypothesis*). Additionally, we assumed that the framework group was able to identify learning strategies more quickly (*Accessibility Hypothesis*). We also expected that the pre-training intervention made learning more efficient (outcome/effort) because of reduced cognitive disorientation (*Efficiency Hypothesis*), and that the framework group exerted less (self-reported) mental effort (*Mental-effort Hypothesis*). Due to greater knowledge about primary learning strategies, we also assumed that self-efficacy with respect to diagnosing primary learning strategies was higher in the framework group than the control group (*Self-efficacy Hypothesis*). Finally, we surmised expected that our pre-training intervention enhanced interest (*Interest Hypothesis*).

## Materials and methods

### Ethic statement

We conducted this study in accordance with the German Psychological Society's (DGPs) ethical guidelines (2004, CIII) as well as APA ethical standards. According to the German Psychological Society's ethics commission, approval from an institutional research board only needs to be obtained if funding is subject to ethical approval by an Institutional Review Board. This research was reviewed and approved by the Ministry of Science, Research, and Arts of Baden-Württemberg, Germany (grant number 7532.3/130), which did not require additional Institutional Review Board approval. The Ministry of Science, Research, and Arts of Baden-Württemberg, Germany, approved the research procedures in this study. The participants volunteered and received 15€ as well as a learning environment for participation. All were aware of taking part in research. We read a standardized explanation about ethical guidelines to them, and they all provided verbal informed consent. Participants not willing to provide verbal informed consent could drop out of the investigation immediately and still receive payment. All participants provided written informed consent permitting us to use their data anonymously. All data was collected and analyzed anonymously.

### Participants and design

Fifty-six student teachers (41 female, M*_age_* = 23.82 years; *SD* = 2.02) from the University of Luxembourg participated in this experiment. All were enrolled in a bachelor program in Educational Sciences in order to become teachers. Their average high school grade in mathematics was *M* = 42.22 (*SD* = 7.66) (60-point-scale from 50–60 = *grade A* to 01–09 = *grade F*). Most of the students (82.1%) had never attended courses on learning strategies or diagnosing learning strategies before. Their self-rated knowledge about primary learning strategies was *M* = 48.57% (*SD* = 16.45%, range: 0–80%) on average. Most of the students (92.9 %) had experience writing learning journals [i.e., diaries describing what they had learned that day) that are suitable for assessing learners' learning strategies; for a detailed description see (Glogger et al., 2009)], which we took as an example of applied learning strategies in our learning environment. The experiment was integrated into a course called “Successful teaching and learning strategies in early childhood education.”

Each of the students was randomly assigned to one of two parallel seminars which were then assigned to the framework condition (*n* = 27) or the control condition (*n* = 29). We conducted the experiment at the very beginning of the seminar (in the second lesson; the first lesson was an organizational lesson). Both seminars were guided by the same teacher, and the contents were identical. Dependent variables were the students' learning outcomes as measured by their ability to define the concept of primary learning strategies, the number of identified learning strategies in excerpts of their learning journals; the *quality of explanations* as to why the identified primary learning strategy belongs in that category; the *accessibility* of learning as measured by the time needed to categorize activities as either primary learning strategies or another category. Furthermore, we calculated the ratio of the post-tests core to the learning time needed for the pre-training intervention and the learning environment as a measure for *efficiency* (Hoffman and Schraw, 2010). Moreover, we measured topic-specific interest and self-efficacy as dependent variables.

### Materials

#### Pretest

A pretest assessing prior knowledge consisted of a self-assessment measure (11-point scale from 0: *very low*, to 100: *very high*) and a direct measure of prior knowledge. The direct measure included the following three open-ended questions: (1) “What do you understand learning strategies to be?” Please provide a definition that includes the typical characteristics of a learning strategy, (2) “What are important learning strategies in your subjects? In which way do they support learning?” and (3) “Do you know the difference between primary and secondary learning strategies? If yes, what are the main differences between primary and secondary learning strategies?” We assessed prior knowledge via a self-developed coding scheme. The definition of learning strategies (question 1) should include the following three aspects: (Primary) learning strategies are (a) cognitive processes, (b) learner-initiated, (c) help construct knowledge. For each of these components the participants could earn 0.5 (implicitly mentioned) or 1 (explicitly mentioned) point so that they could score a maximum of three points for the first question. The strategies mentioned in the second question were scored if they were consistent with the three characteristics of learning strategies or the taxonomy of, for example, Weinstein and Mayer (1986) or related models (e.g., Bråten and Strømsø et al., 2003; Strømsø et al., 2003). This taxonomy is also used in numerous textbooks in a context of educational science, educational psychology, and pedagogy applying to teacher education (e.g., McCormick and Pressley, 1997; Krapp and Weidenmann, 2001; Martinez-Pons, 2001; Landmann et al., 2009; Klauer and Leutner, 2012). Additionally, we found several practitioner-oriented publications that provide learning-strategy instruction referring to the taxonomy of Weinstein and Mayer (1986) such as an established training program for developing reading strategies (“Text Detectives”; Gaile et al., 2007). Each correct strategy was awarded one point. There was no maximum number of points for the second question. In the third question, an entirely correct answer was given one point, “implicitly” correct answers got half a point (maximum = 1). Two independent raters scored the student teachers' answers to the three open-ended questions (not adjusted *ICC = 0.81*).

#### Pre-training intervention

To conceptualize the pre-training intervention, we reanalyzed data originally used for controlling differences in prior knowledge in previous studies (Glogger et al., 2011, 2012). Furthermore, we considered findings from U.S. studies (Clift et al., 1990; Hamman, 1998). As scientific knowledge we applied frequently-cited taxonomies such as that by Weinstein and Mayer (1986) and related approaches. On this basis, we identified four key categories which (student) teachers often confuse with primary learning strategies: secondary learning strategies (e.g., creating a time table), problem-solving strategies (e.g., writing out what is known and sought), using tools (e.g., note cards), and teacher-directed strategies (e.g., group work). These four categories share some but not all defining features of learning strategies.

Both groups underwent a computer-based pre-training intervention in which we accentuated the difference between primary learning strategies and the four similar categories we had identified. Hence, in the pre-training intervention we elaborated on strategies related to but significantly different from primary learning strategies as preparation for the learning environment in which we elaborated on primary learning strategies. An overview of the pre-training interventions for the framework and the control group provides Supplementary Table 1. In these pre-training interventions we first informed all student teachers about assessing their students' learning strategies (*informed training*, Paris et al., 1982). Afterwards, both groups received a brief definition of primary learning strategies according to Weinstein and Mayer (1986): “A primary learning strategy is a mental process that is directly referred to learning, learner-initiated and serves to construct knowledge.” Then, we provided both groups with an introduction about learning journals. We included this introduction because the pre-training intervention and learning environment contained excerpts from learning journals to illustrate the applied learning strategies. These parts of the pre-training intervention were identical for both groups. The pre-training interventions differed in the next step: in the framework group, we stated that primary learning strategies are often confused with other strategies. In the control group we stated that assessing primary learning strategies is sometimes difficult. We then handed each group the same excerpt from a learning journal in which a student applies a primary learning strategy (elaboration) and explains what makes this a primary learning strategy (“if a student develops her own thoughts, a primary learning strategy is being applied. She is dealing cognitively-actively with the learning content and has constructed knowledge”). Next, both groups were given corresponding examples from each of the four similar categories (using excerpts from learning journals) and an explanation as to why these are not primary learning strategies. At that phase, what differed between the experimental and control groups was that we made the framework group aware of the similar categories (e.g., “primary learning strategies can be confused with problem-solving strategies”) as a basis for rejecting intuitive ideas of learning strategies (inappropriate pieces of knowledge). Only the framework group received additional information about which category the non-primary strategies belonged to, while the control group did not. For example, regarding the similar category “problem-solving strategies” we told the framework group: “If a student is deliberating on what has been provided and on what he is being asked to do, he is not applying a learning *but rather a problem-solving strategy*, since that procedure primarily helps to solve the problem.” We told the control group: “If a student is deliberating on what has been provided and on what he is being asked to do, he is not applying a learning strategy since that procedure primarily helps to solve the problem.” Hence, student teachers in the control group received the same factual information, but no framework explaining the strategy categories that are often mistaken for primary learning strategies. Omitting the categorical information was the main difference between the control and framework groups. This “categorical information” is somehow comparable to categorical refutational text (e.g., Skopeliti and Vosniadou, 2008; Tippett, 2010; Sinatra and Broughton, 2011): the related strategy was refused to be a primary learning strategy (for both groups) but only the framework group received the information to which category the related strategy belonged instead. Hence, the framework group acquired *negative knowledge* (e.g., Gartmeier et al., 2008) that is knowledge about which pieces of knowledge are incompatible and should be avoided during later learning in the learning environment. The control group did not receive such negative knowledge about primary learning strategies.

In both conditions we presented specific prompts that guided the learners to focus on the central concept of the pre-training intervention. This is how we encouraged the student teachers to study the pre-training contents more intensely. These prompts were either identical or similar for both groups as far as possible. One example of a prompt is: “What is the most important thing you have learned about primary learning strategies? What did you find particularly hard to understand?” For the framework group, an overview of the different categories concluded the pre-training intervention. The control group received an overview of the different examples concluding the pre-training intervention. In addition, structure, colors, and the number of presented pages (25 PowerPoint slides) of the pre-training interventions were identical for both groups. Consequently, the framework and control groups received similar materials in their pre-training interventions.

#### Learning environment of primary learning strategies

After the preparatory pre-training intervention, the student teachers learned with a computer-based learning environment on how to classify primary learning strategies (taxonomy by Weinstein and Mayer, 1986). In the learning environment, excerpts of learning journals are used as examples for the primary learning strategies. The main categories organization, elaboration, and metacognition and their assessment were explained. We did not limit learning time. A detailed description of the learning environment is provided by Glogger et al. (2013).

#### Interest

We measured situational interest by six items (Magner et al., 2014) on a 6-point Likert-scale (6: *absolutely true*, Cronbach's α = 0.90). Three items were emotion-related (e.g., “It was entertaining to learn how to assess primary learning strategies”) and three items were value-related (e.g., “Learning how to assess primary learning strategies is important”).

#### Self-efficacy

We measured self-efficacy using five items constructed according to Bandura's guidelines (1989). The participants rated how they expect to manage different tasks concerning primary learning strategies (i.e., assessing primary learning strategies in learning journals; distinguishing different primary learning strategies; teaching primary learning strategies; fostering students' primary learning strategies; distinguishing primary learning strategies from similar strategies; Cronbach's α = 0.89) on a 11-point scale from 0% (*not at all*) to 100% (*absolutely*).

#### Post-test

In the post-test we measured the student-teachers' knowledge about primary learning strategies. The post-test consisted of four types of tasks to measure different types of knowledge: a definition task, identification and explanation tasks, and verification tasks. Items in the post-test covered only learning contents from the learning environment.

In the *definition task* we asked the student teachers to define primary learning strategies (as already done in the pretest; see the Pretest section). We used the score in this task as a measure for the student-teachers' knowledge about the concept of primary learning strategies (definition). Two independent raters scored the level of comprehension in the students' answers to the open-ended questions (not adjusted *ICC* = 0.90).

In the *identification task*, student teachers had to identify primary learning strategies in five post-test items. These items were different excerpts from a learning journal in which one or several primary learning strategies had been included. We analyzed answers to open-ended post-test items using a self-developed coding scheme. We gave one point if the teachers mentioned learning strategies related to the main categories (i.e., rehearsal, organization, elaboration, metacognition) provided in the learning environment (Cronbach's α = 0.65). Two independent raters scored 20% of the post-test answers (not adjusted *ICC* = 0.99). We used this task's score as a dependent variable for the ability to identify primary learning strategies.

Additionally, the student teachers were asked to explain their decisions. We measured the *quality of explanations* by a self-developed coding scheme (capacity, consistency, and closure; Biggs and Collis, 1982). Two independent raters scored 20% of the post-test answers (not adjusted *ICC* = 0.98). This measure displayed a low reliability estimate in terms of internal consistency (Cronbach's α = 0.55). However, Schmitt (1996) warned against potential losses of eliminating measures with lower reliability scores because even measures with low alpha levels can be quite useful (we take up this issue in the Discussion). We used this score to operationalize the quality of explanations.

In the *verification task* student teachers were asked to decide as quickly as possible whether or not a presented activity was a primary learning strategy [46 items, Cronbach's α = 0.83, e.g., generating an example (primary learning strategy); using a calculator (not a primary learning strategy)]. Each item could be classified into one of the categories presented in the pre-training intervention. We measured the accessibility of learning by the number of correctly classified learning strategies in the verification task divided by the time needed to do this assignment.

Moreover, we computed as a dependent variable an aggregated measure of *efficiency*. Efficiency was computed as the learning outcome (sum of z-standardized values for correctly identifying learning strategies and the quality of the explanation in the identification task plus the identified learning strategies in the verification task) divided by the time needed in the pre-training intervention and the learning environment (Hoffman and Schraw, 2010).

#### Mental effort

Mental effort is defined as the aspect of cognitive load that learners need to solve a task. It thus represents the actual cognitive load while solving a task (Paas et al., 2003). We used subjective ratings of task difficulty as a measure of mental effort (Paas and van Merriënboer, 1993, 1994). We measured the perceived mental effort twice: after working with the pre-training intervention (Cronbach's α = 0.71) and after working with the learning environment (Cronbach's α = 0.78). Each scale consisted of four items on a 7-point Likert-scale and was adapted to the respective tasks (7: I fully agree, e.g., “it took me a lot of effort to understand what primary learning strategies are”).

### Procedure

The study was embedded in two regular seminars for student teachers to which they were assigned randomly. The framework and the control groups completed the study on two different days (Tuesday and Wednesday) at the same time. First we assessed participants' prior knowledge about learning strategies as well as their self-rated prior knowledge. Next, the student teachers received the condition-specific pre-training intervention (*M* = 21.12 min, *SD* = 4.85 min). They then worked in the computer-based learning environment (*M* = 17.49 min, *SD* = 6.44 min). Then we assessed the student teachers' topic-specific interest. The student teachers completed the post-test. The entire procedure was computer-based. The student teachers needed an average 64.47 min (*SD* = 9.66 min) to complete the experiment.

## Results

Table 1 displays the means and standard deviations of key variables in both conditions. An alpha level of 0.05 was used for all statistical tests. As an effect size measure, we used partial η^2^ Cohen (1988)—qualifying values < 0.06 as small effects, values in the range between 0.06 and 0.13 as medium effects, and values > 0.13 as large effects.

**Table 1:** **Means (and standard deviations) of learning outcome measures**.

	**Framework group *(N* = 27)**	**Control group *(N* = 29)**	**η^2^**
	***M (SD)***	***M (SD)***	
Pretest	1.41 (1.94)	1.00 (1.12)	0.01
Self-rated prior knowledge (%)	47.78 (18.05)	49.31 (15.10)	0.00
**TIME (in minutes)**			
Pre-Training	23.27 (3.70)	19.20 (4.19)	0.18^***^
Learning environment	17.70 (3.55)	17.29 (3.37)	0.00
Verification task	4.42 (1.04)	5.45 (1.99)	0.10^**^
**MENTAL EFFORT**			
Pre-training	3.10 (0.68)	3.16 (0.93)	0.00
Learning environment	2.88 (0.79)	3.00 (0.99)	0.01
**LEARNING OUTCOMES**			
Definition of learning strategies	1.91 (0.84)	1.28 (0.58)	0.17^**^
Identification of learning strategies	5.70 (1.79)	4.59 (1.82)	0.09^*^
Quality of explanation	0.82 (0.34)	0.65 (0.31)	0.07^*^
Verification task	36.93 (5.36)	35.21 (4.70)	0.03
Accessibility (correct items in the verification task/ task-duration)	0.15 (0.04)	0.12 (0.04)	0.09^*^
Efficiency (learning outcome/learning time)	0.11 (0.41)	−0.10 (0.38)	0.07^*^
Interest	5.27 (0.78)	4.84 (0.95)	0.06^*^
Self-efficacy (%)	61.85 (14.57)	54.00 (15.58)	0.07^*^

* p < 0.05;

** p < 0.01;

*** *p < 0.001*.

### Pre-analysis

Groups did not differ in prior knowledge or demographic variables such as age, grades in mathematics and A-levels (high school GPA), teaching experiences, numbers of semesters, mother tongue, and general computer skills (all *p*s > 0.10). The materials were provided in German. In the framework group, all participants spoke Luxembourgian (very similar to German) as their first language, whereas all but five participants in the control group had German or Luxembourgian as the mother tongue. More participants in the control group reported having experience with assessing learning strategies (27.6 vs. 7.4% in the framework group). None of the variables mentioned in this paragraph were predictors for learning outcomes.

With respect to prior knowledge, the overall mean was 1.20 (*SD* = 1.57), that is, very low. 39.3% of the student teachers did not score any points in the pretest. The median was 1. In the first question (definition of primary learning strategies), the mean score was *M* = 0.44 (*SD* = 0.67) (maximum score for this question was 3 points). In the second question (examples of primary learning strategies) the mean score was *M* = 0.70 (*SD* = 1.06) (no maximum score, one point for each correct strategy). In the third question (difference between primary and secondary learning strategies) the mean score was *M* = 0.06 (*SD* = 0.23) (maximum score for this question was 1 point). The pretest scores were not predictive for any outcome variables, presumably due to floor effects. It thus made no sense to use prior knowledge as a covariate in further analyses.

We also analyzed student teachers' knowledge that was incompatible with the normative knowledge in terms of the four similar categories. Overall the student teachers mentioned on average 1.16 similar categories in the pretest. The median was 1.00. Only 26.8% mentioned no similar category. In greater detail: 28.6% mentioned at least one secondary learning strategy, 10.7% using tools once or more often, 32.1% had at least one problem-solving strategy, and 33.9% at least one teaching strategy. There were no differences between groups with respect to the aforementioned similar categories.

Student teachers in the conditions did not differ in the time spent in the learning environment, however, the framework group worked with the pre-training intervention significantly longer (*M* = 23.27 min, *SD* = 3.70 min) than the control group (*M* = 19.20 min, *SD* = 4.19). We considered the time spent in the pre-training intervention when calculating the efficiency measure (for a more detailed description see the method section).

### Hypotheses testing

To test our hypotheses on the pre-training effects, we conducted *t*-tests (one-tailed). In the post-test, the framework group mentioned significantly more main criteria of primary learning strategies (ability to define primary learning strategies) than the control group, *t*_(54)_ = −3.29, *p* = 0.001, η^2^ = 0.17. Our Definition Hypothesis was thus confirmed.

The framework group significantly outperformed the control group in their ability to identify primary learning strategies, *t*_(54)_ = −2.31, *p* = 0.01, η^2^ = 0.09. We thus also confirmed the Identification Hypothesis. Moreover, the framework group significantly outperformed the control group in the quality their explanations about whether or not something was a primary learning strategy, *t*_(54)_ = −1.93 *p* = 0.03, η^2^ = 0.07, confirming the Explanation Hypothesis as well. In the verification task, the framework and the control group did not significantly differ in correctly assigning primary learning strategies *t*_(54)_ = −1.28, *p* = 0.10, η^2^ = 0.03.

Furthermore, we measured the accessibility of knowledge about primary learning strategies by calculating the number of correctly-categorized items (primary learning strategy vs. no primary learning strategies) in the verification task divided by the time spent on this task. The framework group outperformed the control group concerning accessibility, *t*_(54) = −2.29_, *p* = 0.01, η^2^ = 0.09. That is, the framework group categorized an approximately equal number of items correctly in less time, *t*_(43)_ = 2.46 *p* < 0.01, η^2^ = 0.10, thus confirming the Accessibility Hypothesis.

We determined the efficiency measure of learning as the ratio of the learning outcome divided by learning time in the pre-training intervention and learning environment. We identified an extreme value in one participant for the time spent in the learning environment. We changed this score to fall one unit above the next-highest score in the data set (Field, 2009). The data on the time in the pre-training intervention and the learning environment were not normally distributed. We therefore transformed the data logarithmically to lessen the impact of outliers and skew (Whelan, 2008). We computed the measure of the learning outcomes out of z scores for the identification measure, the explanation measure, and the number of correctly identified strategies in the verification task. In learning-efficiency terms, the framework group also outperformed the control group, *t*_(50) = −1.87_, *p* = 0.03; η^2^ = 0.07. The framework group revealed higher learning outcomes, *t*_(54)_ = −2.62, *p* = 0.01, η^2^ = 0.09 although they spent significantly more time in the pre-training intervention and in the learning environment, *t*_(50)_ = −2.84, *p* < 0.01, η^2^ = 0.14. Hence, our Hypothesis of Efficiency was confirmed. However, this difference was only due to higher learning outcomes.

We detected no significant differences between the framework and control groups in the mental effort perceived during the pre-training intervention, *t*_(54)_ = 0.24, *p* = 0.40, η^2^ = 0.00 and during the learning environment *t*_(54)_ = 0.54, *p* = 0.29, η^2^ = 0.01. Thus, the Mental-effort Hypothesis was thus not confirmed.

The participants' self-efficacy in working with the concept of primary learning strategies was on average *M* = 57.79% (*SD* = 15.48). The framework group claimed to have significantly higher self-efficacy values after working with the learning environment, *t*_(54)_ = −1.94, *p* = 0.03, η^2^ = 0.07 (Table 1), thus confirming the Self-efficacy Hypothesis. The student teachers' interest in assessing primary learning strategies was relatively high overall after working in the learning environment (*M* = 5.05, *SD* = 0.88, scale from 1 = *low* to 7 = *high*). Nevertheless, participants in the framework condition were significantly more interested in learning strategies after working in the learning environment *t*_(54)_ = −1.83, *p* = 0.04, η^2^ = 0.06 (Table 1). We therefore confirmed the Interest Hypothesis.

For explorative reasons we analyzed the frequency and type of similar categories in all post-test tasks. In so doing we observed that the framework group mentioned significantly fewer similar categories than the control group, *t*_(54)_ = 1.67, *p* = 0.05, η^2^ = 0.05. More specifically, they mentioned fewer teaching strategies, *t*_(54)_ = 2.43, *p* = 0.005, η^2^ = 0.10 and fewer problem-solving strategies, *t*_(54)_ = 1.93, *p* = 0.03, η^2^ = 0.07.

## Discussion

We propose the instructional approach of providing a categorical framework in order to enable learners with prior knowledge in pieces to learn more, more intensively, and more efficiently. Our findings demonstrate beneficial effects of the framework we provided on all outcome measures except mental effort. The student teachers in the framework group were better able and equipped to provide a definition of primary learning strategies (*Definition Hypothesis*, large effect) and to identify primary learning strategies correctly (*Identification Hypothesis*, medium effect). Furthermore, student teachers in the framework group gave better explanations (*Explanation Hypothesis*, medium effect) which can be considered an indicator of deeper knowledge. Moreover, student teachers in the framework group were able to access their knowledge about primary learning strategies more quickly (*Accessibility Hypothesis*, medium effect). Furthermore, they learned more efficiently (*Efficiency Hypothesis*, medium effect) in that they displayed a significantly better learning outcome (although needing more time than the control group). However, as just mentioned, we detected no significant differences in mental effort (*Mental-effort Hypothesis*). We found the expected positive effects from the categorical pre-training intervention on the motivational variables. Student teachers in the framework group claimed to possess both higher levels of self-efficacy and interest (*Self-efficacy Hypothesis* and *Interest Hypothesis*, both medium effects) to assess their students' primary learning strategies.

(Student) teachers' professional knowledge about primary learning strategies implies that they are capable of distinguishing primary learning strategies from one another and from similar strategies. Furthermore, (student) teachers need to be aware of the different functions of learning strategies, and the specific contexts in which they should be applied. The student teachers in the framework group learned more and in greater depth about primary learning strategies and could assess this knowledge faster than the control group. This is an important finding, because well-organized knowledge can be regarded as a prerequisite for teachers to be able to assess and foster learning strategies in their classrooms.

Gaining appropriate knowledge about learning strategies is just the first step toward (student) teachers applying this knowledge in classrooms. However, we found that our framework group was significantly more interested in assessing primary learning strategies and that they claimed to possess higher levels of self-efficacy in that regard. Higher levels of both self-efficacy and interest can increase the probability that learners will apply the knowledge they have acquired (Renkl et al., 1996).

The effect we observed on teachers' efficiency is in line with our assumption that our abstract, categorical scheme served as a cognitive framework upon which the student teachers could reorganize their prior knowledge. This framework might have suggested to them a new way of restructuring their prior knowledge and of “moving” pieces of knowledge that were incompatible with the scientific knowledge at hand to categories other than primary learning strategies (secondary learning strategies, using tools, problem-solving strategies, and teaching strategies). Such reorganized prior knowledge may have reduced conceptual cognitive disorientation while learning about primary learning strategies. However, this is a tentative explanation that cannot be specifically addressed in this study. Future studies should include an analysis of the topic domain, namely how knowledge that is incompatible with the normative knowledge is based on fragmented knowledge and how increased coherence is what is required.

We did not observe differences in the student teachers' perceived mental effort comparing both groups. However, this does not necessarily imply that there were no differences in their intrinsic cognitive load. It might well be that the intrinsic cognitive load decreased. The learners however could have used their freed cognitive resources to change their concept. The framework group achieved better learning outcomes and, in part, conceptual reorganization. Conceptual restructuring processes, however, accompany high mental effort. Hence, possible effects of reduced mental effort due to the pre-training intervention might were disguised by mental load caused by conceptual change processes and resulted in better knowledge. For example, Muller et al. (2008) found that students who perceived information involving alternative conceptions reported greater mental effort but also attained higher post-test scores than students receiving standard material. They assumed this cognitive load to be a necessary, learning-related load to achieve conceptual change. However, unless we have a valid measure for intrinsic cognitive load we do not know if intrinsic cognitive load is a significant factor in the present study.

Our pre-training intervention provides learners with information on a higher level of abstraction in order to support the organization of knowledge. How can our approach be embedded in the existing body of research concerning prior knowledge? According a broader view of advanced organizers our pre-training intervention can be seen as a type of *advance organizer*. More recently an advanced organizer was conceptualized as a “(…) vehicle for suggesting an appropriate scheme for a reader to access (…)” (Corkill, 1992, p. 40; Gurlitt et al., 2012), the refutational text elements provided in the pre-training intervention can also be seen as a kind of advance organizer (Sinatra and Broughton, 2011). However, according to a traditional view of advance organizers (e.g., Ausubel, 1960) there are three main differences between our pre-training intervention and a classical advance organizer. (1) Traditional (“expository”) advance organizers (Ausubel and Fitzgerald, 1961) activate prior knowledge. Our pre-training intervention activates prior knowledge but likewise aims to change prior knowledge. (2) Traditional advance organizers are directly related to the following learning contents. Our pre-training intervention, however, provides information about contrasting concepts in order to render the relevant concept (primary learning strategies) more salient and is therefore only related indirectly to the learning content (hierarchical and lateral concepts; somewhat similar to comparative advance organizers, e.g., Ausubel and Fitzgerald, 1961). (3) We present learners with categorical refutational elements which advance organizers do not. In a nutshell, our pre-training intervention can in a broader view be seen as a type of advance organizer.

### Limitations and alternative explanations

Despite our promising study results here there are limitations that need to be addressed. One limitation concerns our assessment of the student teachers' explanations. We noted a less reliable score for this measurement (Cronbach's α = 0.55). However, we identified very consistent effects on all different learning-outcome aspects. Thus, we assume that the effect on the explanation quality can be interpreted (although with some caution). In addition, Schmitt (1996) argued that measures with lower alpha levels can also be quite useful.

Although the pretest scores were not significantly different (at *p* = 0.43) the mean pre-test scores differed slightly in favor of the experimental condition. However, the correlations between the pre-test score and any of the outcome variables ranged between *r* = −0.19 and *r* = 0.23 and failed to attain statistical significance. Hence, it is very unlikely that the effects noted in this study are due to differences in prior knowledge between the conditions.

A further study limitation is that we did not limit learning times. Student teachers in the framework condition invested significantly more time than the control group on the task in the pre-training phase. We thus need to consider that effects could have been caused by time spent in the pre-training intervention. However, when we included the time spent in the pre-training intervention as a covariate we still observed significant effects in conjunction with the condition in terms of our learning-outcome measures. Furthermore, we calculated the efficiency measure considering learning time in the pre-training intervention. The framework group still outperformed the control group when the ratio between learning outcome and learning time is compared.

Moreover, further research is needed to verify whether providing an abstract, categorical schema as cognitive framework also has long-term effects on learning. Nevertheless, the effects on the motivational variables (interest and self-efficacy) could be interpreted as fostering the applicability and transfer of knowledge so that long-term effects might be possible.

Furthermore, we only tested the approach of providing a categorical framework on teachers' knowledge about primary learning strategies. Our study findings alone do not allow us to generalize across other learning contents. However, there is evidence that individual components of our pre-training intervention—namely, providing an abstract schema and categorical information—also work in different knowledge domains. For example, provision of an abstract scheme was suitable for technical areas (e.g., function-process-structure schema, Kalyuga et al., 2010; Kalyuga and Hanham, 2011; Kalyuga, 2013) and for biological knowledge (Hmelo-Silver and Pfeffer, 2004). Still, further research should address whether an abstract cognitive framework also works in other knowledge domains.

## Conclusion

The present approach combines an intervention form (pre-training) inspired by research on pre-training and on knowledge in pieces. Until now, research has rarely focused on concrete instructional procedures to address fragmentary knowledge. Overall, this study demonstrates that providing a general scheme can be a promising approach to overcome the barrier of (partly) incorrect prior knowledge in pieces. Moreover, it expands the research on pre-training interventions as inspired by cognitive load theory to domains in which learners have incompatible intuitive knowledge.

### Conflict of interest statement

The authors declare that the research was conducted in the absence of any commercial or financial relationships that could be construed as a potential conflict of interest.
