# Supplementary material for: Preparing learners with partly incorrect intuitive prior knowledge for learning
Source: Front Psychol. 2014 Jul 3;5:664. doi: 10.3389/fpsyg.2014.00664 (PMC4080464; doi:10.3389/fpsyg.2014.00664)
Supplement: Supplementary file 1 [file DataSheet1.DOCX]

**Table 1. Overview of the pre-training interventions for the framework and the control group**

| **Section of the pre-training intervention** | **Framework group** | **Control group** |
| --- | --- | --- |
| Definition of primary learning strategies | 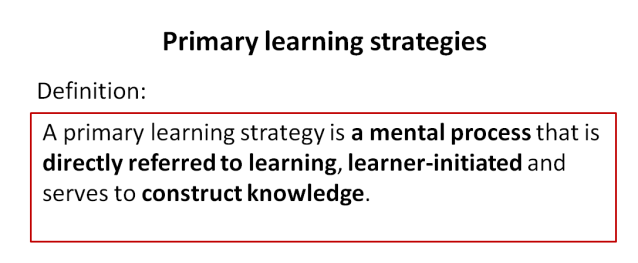 | 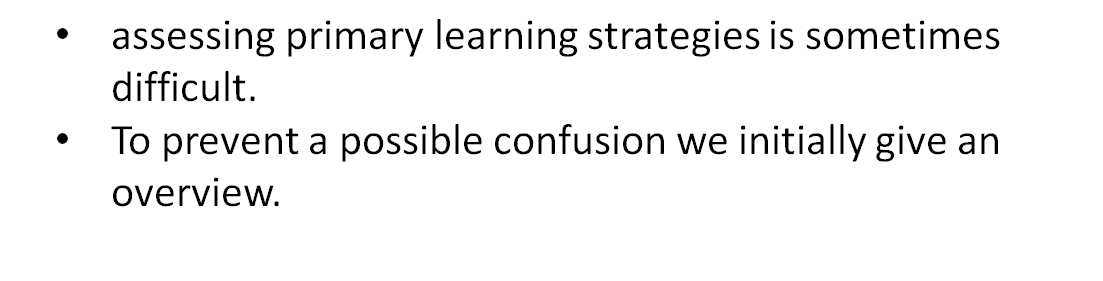 |
|  | 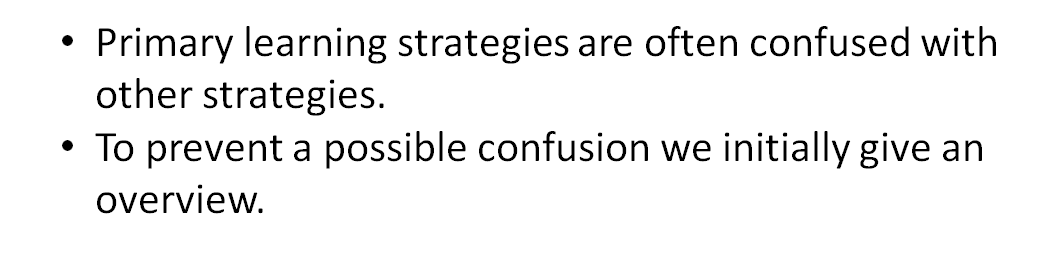 |  |
| Excerpt from a learning journal in which a student applies a primary learning strategy (elaboration) | 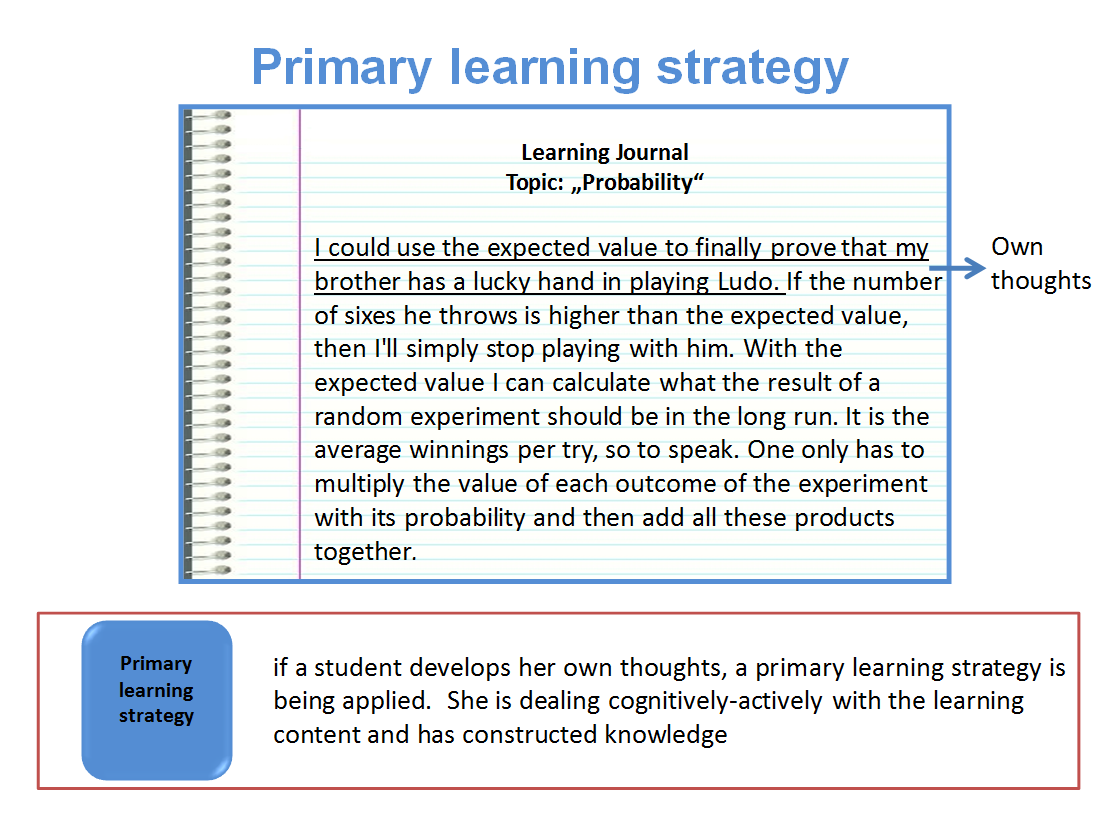 |  |
| Excerpt from a learning journal in which a student applies a problem-solving-strategy | 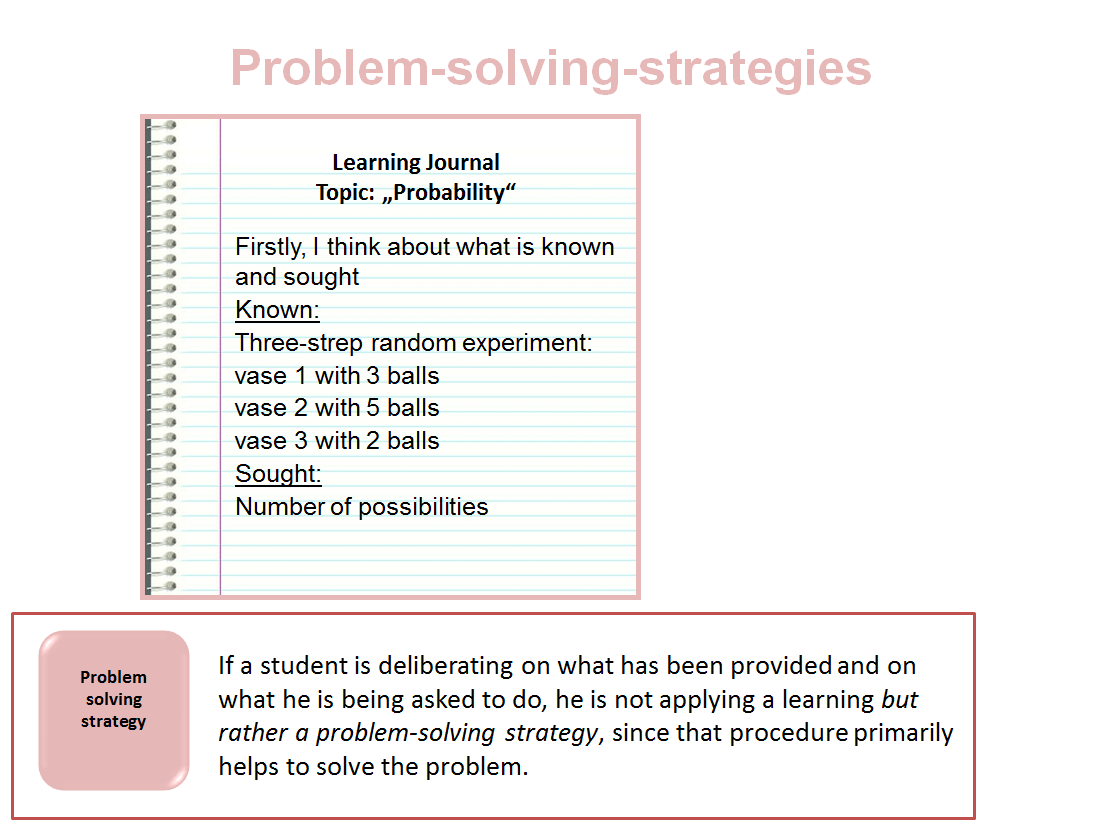 | 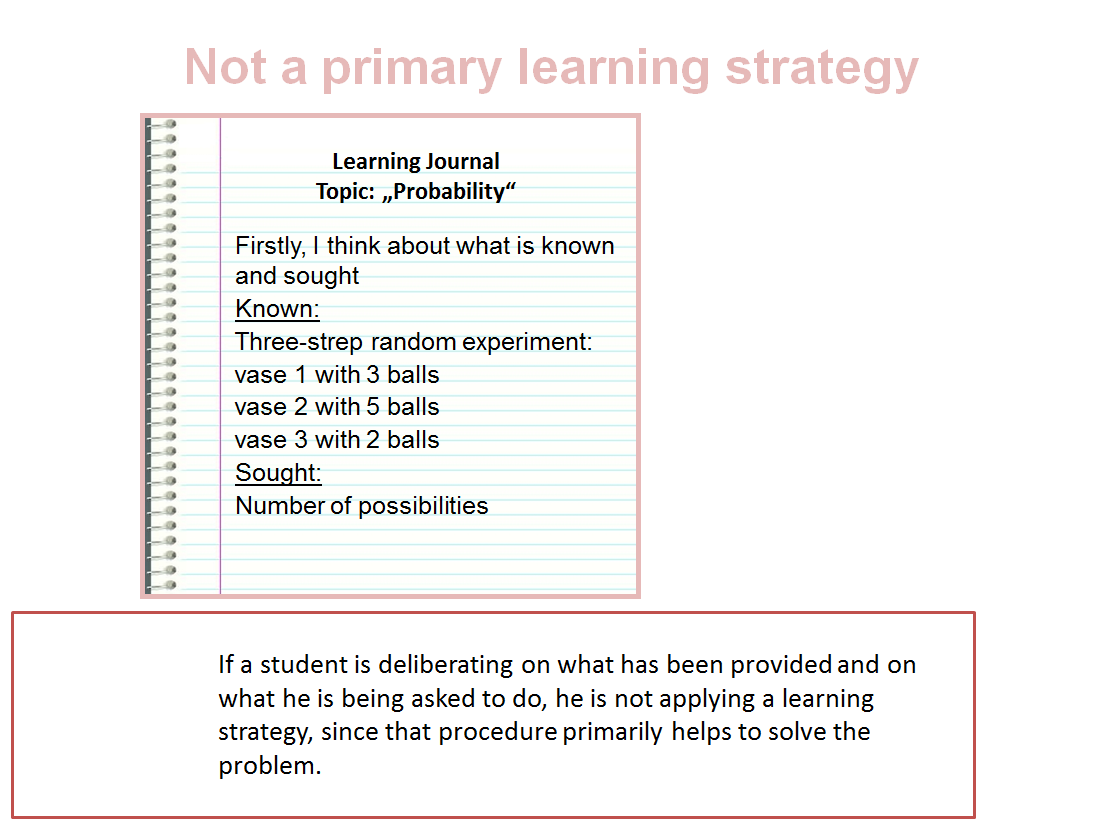 |
| Overview | 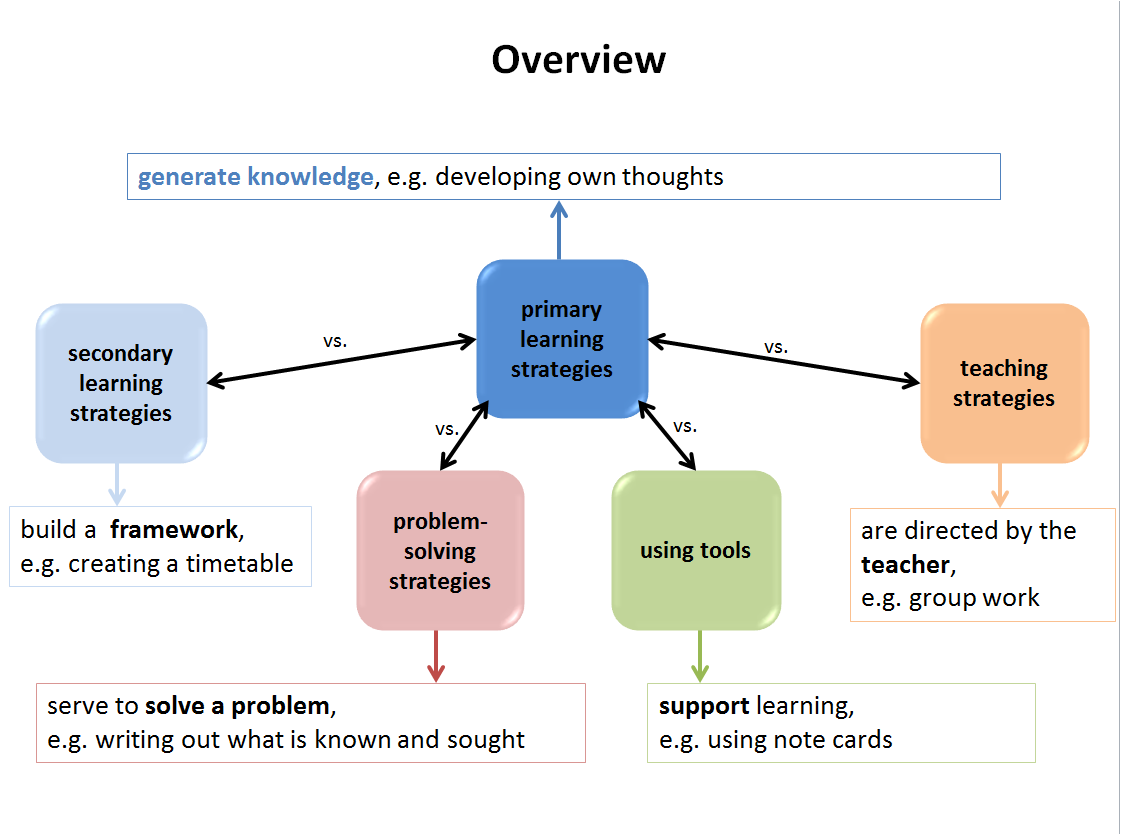 | 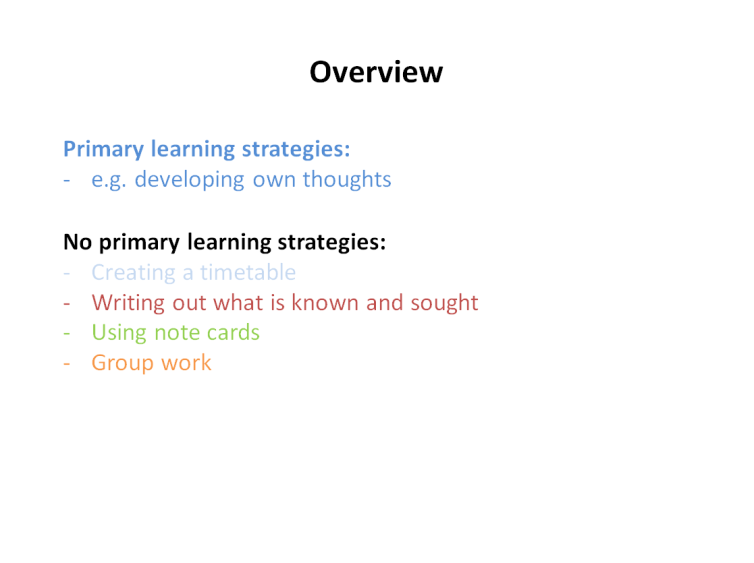 |
